# Defense Peptides From the α-Hairpinin Family Are Components of Plant Innate Immunity

**DOI:** 10.3389/fpls.2020.00465

**Published:** 2020-04-23

**Authors:** Anna A. Slavokhotova, Eugene A. Rogozhin

**Affiliations:** ^1^M.M. Shemyakin and Yu.A. Ovchinnikov Institute of Bioorganic Chemistry, Russian Academy of Sciences, Moscow, Russia; ^2^Chumakov Federal Scientific Center for Research and Development of Immune-and-Biological Products of Russian Academy of Sciences, Moscow, Russia; ^3^All-Russian Institute of Plant Protection, St. Petersburg-Pushkin, Russia; ^4^Gause Institute of New Antibiotics, Moscow, Russia

**Keywords:** antimicrobial peptides, α-hairpinins, plant innate immunity, defense peptides, primary and spatial structures, protein-precursors

## Abstract

Plant immunity represents a sophisticated system, including both basal and inducible mechanisms, to prevent pathogen infection. Antimicrobial peptides (AMPs) are among the innate immunity components playing a key role in effective and rapid response against various pathogens. This review is devoted to a small family of defense peptides called α-hairpinins. The general characters of the family, as well as the individual features of each member, including biological activities, structures of precursor proteins, and spatial structures, are described. Possible applications of α-hairpinin peptides in drug design are discussed.

## Introduction

Constantly attacked by various pathogens, plants developed a sophisticated innate immune system that contains numerous secondary metabolites, antimicrobial proteins, and peptides. Antimicrobial peptides (AMPs) are intensively studied groups of short defense molecules found ubiquitously in microorganisms, fungi, plants, and animals. AMPs represent “the first line of defense,” they expressed continuously or in response to pathogenic attacks (Egorov and Odintsova, 2012). Despite of the wide diversity of AMPs, several significant properties are considered general for all AMPs, including low molecular mass (up to 10 kDa), antimicrobial activity, positive charge, and amphiphilic structure. AMPs exist in various molecular forms: linear peptides usually occur in insects and animals, although cysteine-rich peptides with multiple disulfide bonds are mainly found in plants and bacteria (Tam et al., 2015). Plant AMPs exhibit extreme structural heterogeneity; they share low amino acid sequence similarity, various spatial structures, and a wide range of defense-related properties. The general classification is based on the number of cysteine residues and their molecular arrangement within the so-called cysteine motif (Cys-motif). The main families of plant AMPs include defensins, thionins, lipid-transfer proteins, α-harpinins, hevein-like peptides, snakins, knottins, and cyclotides (Broekaert et al., 1997). Plant AMPs exhibit various biological activities, including antibacterial, antifungal, antiviral, enzyme-inhibitory, cytotoxic, and insecticidal activities (Nawrot et al., 2014). The mechanism of action has been described for a limited number of AMP families; for example, hevein-like peptides bind chitin polymers into the fungal cell wall (Slavokhotova et al., 2017a), knottins inhibit some proteases, and lipid transfer proteins bind lipids to disrupt microbial penetration into cell membranes (van der Weerden et al., 2013).

Plant AMPs are ubiquitous; they are found in all plant organs throughout their lifetime (Egorov and Odintsova, 2012). Based on the analysis of rice and Arabidopsis genomes, a number of cysteine-rich peptides have been found, accounting for about 3% of the gene repertoire (Silverstein et al., 2007). Moreover, transcriptomic analysis of wild-growing plants and weeds has shown that plants contain hundreds of AMP-like sequences (Slavokhotova et al., 2015, 2017b). However, it should be noted that the expression of these genes and the synthesis of functional AMPs have been confirmed for only a limited number of peptides (Marcus et al., 1999; Slavokhotova et al., 2014b). In this mini-review, we describe the main characteristics of α-harpinin peptides. Their unusual gene structures, simple structural conformations, and potent antimicrobial activities make these peptides of great interest for biotechnology and fundamental studies.

## Diversity of α-Hairpinins and Their Biological Activity

α-Hairpinins represent a small family of short peptides that are distinguished from other AMPs by a peculiar Cys-motif (XnC1X3C2XnC3X3C4Xn, where X is any amino acid residue except cysteine) that forms a characteristic helix-loop-helix structure. The first representative was isolated in the early 1990s (Duvick et al., 1992), after which a number of molecules were purified from various monocots and dicots. In Oparin et al. (2012) termed this family α-harpinin peptides; since then, all plant AMPs shared a specific motif formed by four cysteine residues, were called α-harpinins. This AMP family includes highly heterogeneous peptides with a broad spectrum of biological activity: α-hairpinins possessing antifungal and antibacterial activity together with peptides displaying trypsin inhibitory and ribosome-inactivating activity. This part of the mini-review is devoted to the primary amino acid structure of α-hairpinins and their biological activity.

### α-Hairpinins With Antifungal Activity

**FIGURE 1:**
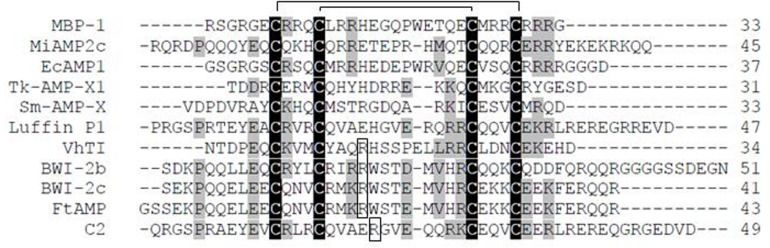
Amino acid sequence alignment of α-hairpinin peptides. Following peptides sequences are shown in alignment: MBP-1 from *Zea mays* (P28794); EcAMP1 from *Echinochloa crus-galli* (P86698); Tk-AMP-X1 (CCP19155.1); Sm-AMP-X (C0HJD6); Luffin P1 from *Luffa aegyptiaca (*P56568); VhTI from *Veronica hederifolia* (P85981); BWI-2b, and BWI-2c from *Fagopyrum esculentum* (no accession number and P86794); C2 peptide from *Cucurbita maxima* (Q9ZWI3). The cysteine residues are shown in gray; disulfide bridges shown in black lines above; the functional for trypsin inhibitors Arg residues are boxed.

The first α-hairpinin was isolated in 1992 by Duvick et al. (1992) from mature maize (*Zea mays*) kernels. Acid extraction and multistage stepwise chromatography resulted in the purification of a small, basic peptide called MBP-1. It was found that the peptide consisted of 33 amino acid residues, and its complete primary structure has been determined by the Edman degradation technique, which showed no homology to any known peptides annotated to AMP families at that time (Figure 1). MBP-1 was found predominantly in the embryo portion of the maize kernels. However, its amount in germinated seeds was rather low (0.014% of total protein), leading to the suggestion that MBP-1 might serve as a major source of rapidly mobilized nitrogen in germinated seeds. *In vitro* antimicrobial assays showed that MBP-1 inhibited spore germination or hyphal elongation of some plant pathogenic fungi, including *Fusarium moniliforme* and *F. graminearum* and it was active against *Escherichia coli* and the bacterial pathogen of maize *Clavibacter michiganense* (Table 1). Based on these data, the peptide was thought to contribute to the resistance of kernels to infection caused by plant pathogenic fungi and bacteria (Duvick et al., 1992).

**TABLE 1:** Diversity of α-hairpinins from plants.

	Peptide name (UniProt/GenBank ID)	Biological activity	Plant source (plant family)	References
1	MBP-1 (P28794)	Antibacterial (*C. michiganense, E. coli)*, antifungal (*F. moniliforme, F. graminearum)*	*Zea mays* (Poaceae)	Duvick et al., 1992
2	MiAMP2c, (Q9SPL5)	Antifungal (*A. alternata*, *C. nicotianae*, *F. oxysporum*, *V. dahlia*, *Ph. cryptogea*, *Ph. parasitica nicotianae, Ch. elegans*)	*Macadamia integrifolia* (Proteaceae)	Marcus et al., 1999, 2008
	MiAMP2b, MiAMP2d	Antifungal (*V. dahlia*, *Leptosphaeria maculans)*		
	MiAMP2a	Antifungal (*B. cinerea*)		
3	Ec-AMP1 (P86698)	Antifungal (*A. alternata, A. solani, Bipolaris sorokiniana, F. graminearum, F. oxysporum, F. solani, F. verticillioides, Phoma betae, Ph. infestans, P. debaryanum*, and P. *ultimum*)	*Echinochloa crus-galli* (Poaceae)	Nolde et al., 2011; Rogozhin et al., 2012, 2018b; Ryazantsev et al., 2014, 2019
	EcAMP1-Hyp	Antifungal (*F. solani*)		
	EcAMP2, EcAMP2.1	Inactive		
	EcAMP3	Antifungal (*F. graminearum, F. oxysporum, A. niger, B. sorokiniana*)		
	EcAMP4, EcAMP4.1	Low trypsin inhibitory activity (EcAMP4), Antifungal (*F. graminearum, F. oxysporum*, and *P. infestan*s)		
5	Tk-AMP-X1 (CCP19155.1), Tk-AMP-X2 (CCP19165.1)	Antifungal (*F. graminearum, F. verticillioides, D. maydis*)	*Triticum kiharae* (Poaceae)	Utkina et al., 2013
6	Sm-AMP-X (C0HJD6)	Antifungal (*A. alternata, B. cinerea, F. oxysporum, F. solani*, and *A. niger*)	*Stellaria media* (Caryophyllaceae)	Slavokhotova et al., 2014b
	Sm-AMP-L, Sm-AMP-X1, Sm-AMP-X2	Antifungal (*A. niger, F. oxysporum, F. solani*)		
7	VhT1 (P85981)	Trypsin inhibitor	*Veronica hederifolia* (Plantaginaceae)	Conners et al., 2007
8	BWI-2a BWI-2b BWI-2c (P86794)	Trypsin inhibitor	*Fagopyrum esculentum* (Polygonaceae)	Park et al., 1997; Oparin et al., 2012
9	FtAMP	Trypsin inhibitor, antifungal (*Trichoderma koningii, Rhizopus* sp., and *F. oxysporum*) *T. koningii, Rhizopus* sp., and *F. oxysporum*	*Fagopyrumtataricum* (Polygonaceae)	Cui et al., 2018
10	C2 (Q9ZWI3)	Trypsin inhibitor	*Cucurbita maxima* (Cucurbitaceae)	Yamada et al., 1999
	6.5k-AGRP, Luffin P1 (P56568)	Ribosome-inactivating	*Luffa cylindrical* (Cucurbitaceae)	Kimura et al., 1997; Li et al., 2003

Marcus et al. (1999) found an antifungal α-hairpinin in *Macadamia integrifolia* (Marcus et al., 1999). The peptide named MiAMP2c was purified from nut kernels (*Macadamia intgrifolia*) and contained 45 amino acid (aa) residues, including four cysteines that formed the Cys-motif (Figure 1). This peptide exhibited antifungal activity against several fungal pathogens at active concentrations of 5–10 μg/ml, including *Alternaria alternata*, *Cercospora nicotianae*, *F. oxysporum*, *Verticillium dahlie*, *Phytophthora cryptogea*, and *Ph. parasitica nicotianae*. The lowest IC_50_ values (2–5 μg/ml) were observed on fungi *Chalara elegans*. This peptide was not active against various bacteria tested (Marcus et al., 1999).

A group of antifungal α-hairpinins was isolated from barnyard grass (*Echinochloa crus-galli*) seeds. The first peptide, called EcAMP1, was purified from crude acidic seed extract (Nolde et al., 2011). It consisted of 37 aa residues and had the characteristic Cys-motif (Figure 1). EcAMP1 inhibited spore germination of various fungi (Table 1) and was extremely active against plant pathogenic fungi from the *Fusarium* genus (*F. graminearum*, *F. solani*, *F. oxysporum*) and against *Phoma betae* with EC_50_ ranging from 1 to 10 μM. The observed activity was comparable to that of MBP-1: the effective concentrations of both peptides against *F. graminearum* were around 4 μM. By light microscope assay, it was revealed that EcAMP1 prevented hyphae elongation without cytoplasmic membrane lysis. Moreover, experiments with *Fusarium* species showed that the peptide did not affect the germination from the conidia itself (Nolde et al., 2011). Accordingly, this was the first plant α-hairpinin demonstrated to have fungistatic activity.

The mechanism of action of EcAMP1 against *F. solani* was further investigated with a combination of classical microbiological approaches and various microscopy techniques (Vasilchenko et al., 2016). Optical microscopy observation revealed a linear correlation between the dose and the response at a concentration of EcAMP1 less than the IC_50_. The antimicrobial effect was more pronounced against germinated conidia than against the ungerminated stage. Using high-resolution laser scanning fluorescence microscopy, an interaction between EcAMP1 and the target cell was observed. At the first stage, the active peptide bound with components of the fungal cell wall (with glycans, glycoproteins, and proteins-amyloids) and distributed uniformly over the entire cellular surface. At the second stage, the peptide expanded in the cell barrier structures uniformly, presumably due to an abundance of binding sites located homogeneously across the plasma membrane and/or cell walls of the conidia surface. Moreover, if the concentration of EcAMP1 was greater than IC_50_, the roughness of the conidia surface increased, and the cell volume decreased in a dose-dependent manner. Perhaps the most plausible mechanism of EcAMP1 action is an induction of apoptosis, leading to fungal programmed cell death, different to the membrane-disruption mechanisms of action of various other plant AMPs (Vasilchenko et al., 2016).

Besides EcAMP1, several peptides with specific α-hairpinin Cys-motifs were purified from barnyard grass (*E. crus-galli*) seeds. The first identified peptide (EcAMP1-Hyp) was a natural analog of EcAMP1 that differed by a modification of the proline to hydroxyproline residue at position 19 (Rogozhin et al., 2018a). This single amino acid substitution resulted in weaker antifungal activity against *F. solani* and reduced binding affinity with commercial polysaccharides, chitin, and β-1.3-glucan *in vitro* (Rogozhin et al., 2018a). EcAMP2 and its truncated analog EcAMP2.1 contained 31 and 26 aa residues, respectively, and were slightly homologous to EcAMP1 (approximately 40% similarity between EcAMP1 and EcAMP2) (Rogozhin et al., 2012). These two peptides equally decreased the growth of zoosporangia of *P. infestans* at a concentration of 24 μM, were not able to inhibit colony growth of any bacterial species tested, and had no trypsin-inhibitory activity (Rogozhin et al., 2012). EcAMP3 has 35 aa residues and shares 40% homology to the EcAMP1 peptide (Ryazantsev et al., 2014). This peptide showed no trypsin inhibitory activity but had a significant inhibitory effect on mycelium growth of some phytopathogenic fungi (Table 1). Unlike EcAMP1 and EcAMP2, EcAMP3 suppressed the growth of bacteria with an IC_50_ ranging between 10 μM (*P. syringae*) and 20 μM (*E. carotovora*) (Ryazantsev et al., 2014). The EcAMP4 peptide and its truncated analog EcAMP4.1 contain 38 and 31 aa residues, respectively, and have low similarity to EcAMP1: they share less than 27% aa identity (Ryazantsev et al., 2019). Contrary to all EcAMPs peptides, EcAMP4 displayed low trypsin inhibitory activity, whereas EcAMP4.1 was not able to inhibit trypsin. EcAMP4 showed high antifungal activity against *F. graminearum*, *F. oxysporum*, and *P. infestans* at a concentration of 8 μM, while EcAMP4.1 was less effective and had an IC_50_ that ranged between 12 and 18 μM. The authors concluded that among all studied EcAMPs, the EcAMP1, EcAMP3, and EcAMP4 peptides have similar activities, peptide EcAMP4.1 was less active, and peptides Ec-AMP2 and EcAMP2.1 were almost inactive (Ryazantsev et al., 2019).

Two α-hairpinins were isolated from seeds of wheat *T. kiharae* and named Tk-AMP-X1 and Tk-AMP-X2 (Utkina et al., 2013). These highly similar molecules contained 31 and 28 aa, respectively, as well as the α-hairpinins Cys-motif. *In vitro*, these peptides inhibited the spore germination at comparable concentrations, however, against some fungi, Tk-AMP-X2 was more active. Peptides effectively inhibited the growth of *F. graminearum* at equal concentrations (IC_50_ = 7.5 μM), but were less active against *F. verticillioides* (IC_50_ ranged from 10 to 15 μM), and had relatively high concentrations against *D. maydis* (IC_50_ between 17 and 30 μM). Neither of the wheat peptides exhibits protease inhibitory activity (Utkina et al., 2013).

Finally, the last representative of α-hairpinins with antifungal activity, Sm-AMP-X peptide, was isolated from chickweed (*Stellaria media*) seeds (Slavokhotova et al., 2014b). Sm-AMP-Xcontains 33 aa residues and a potent antifungal activity, with an IC_50_ that ranges from 4 to 10 μM. Against some fungi, the inhibition was more pronounced with germinated spores, while against others, the persistence effect was observed with ungerminated conidia. Of the all fungi tested, the highest inhibition was observed on *A. niger* with IC_50_ of 3 μM. In comparison to EcAMP1, Sm-AMP-X was less active against *Fusarium* species (Nolde et al., 2011), but also exceptionally fungistatic, because did not lead to fungal plasma membrane disruption.

Based on the Sm-AMP-X amino acid sequence, three recombinant peptides were expressed in *E. coli* culture: the first, Sm-AMP-L, was obtained to optimize purification and final yield of the chickweed α-hairpinin, and two truncated peptides (Sm-AMP-X1 and Sm-AMP-X2) to determine the functional role of the unstructured tail regions. The peptide Sm-AMP-X1 had both disulfide bonds preserved while amino acids in positions 1–8 and 31–33 were removed, and Sm-AMP-X2 contained only the inner S–S-bond remaining (amino acids 1–12 and 27–33 were deleted). The antifungal activity assays show that Sm-AMP-L has biological activity similar to Sm-AMP-X, whereas the truncated Sm-AMP-X1 had significantly decreased antifungal activity and, among all the peptides, Sm-AMP-X2 was the least active. Considering this data and low antifungal activity, it was concluded that the tails of the molecule contributed either through direct interaction with the fungi or through stabilization of the helical structure (Slavokhotova et al., 2014b).

Rogozhin et al. (2018b) identified several structural elements of α-hairpinins that play a key role in their biological activity. According to their experiments and the literature data, the first crucial element is a β-hairpin structure (10–13 amino acid residues) that connects two α-helices. The second element is a mini-cluster of positively charged amino acid residues necessary for interaction with negatively charged carbohydrate components and polymers of fungal cell walls. And the third element is the presence of at least one hydrophobic residue (e.g., tryptophan) pivotal for binding with components of fungal cell membranes, including sphingolipids or ergosterols (Rogozhin et al., 2018b).

### α-Hairpinins With Trypsin Inhibitory Activity

There are several members of the α-hairpinin family possessing trypsin inhibitory activity with a canonical mechanism of inhibition. The peptides accumulate mainly in seeds, and preliminary provide a defense against insect pathogens. According to the mechanism, the inhibitors bind the substrate with a rigid, reactive-site loop that is complementary to the binding site of serine proteinases (trypsin) (Bateman and James, 2011). α-Hairpinins bind the trypsin in the manner of a good substrate, the binding is tight, and it is hydrolyzed very slowly. The “canonical inhibitor loop” is correspondent to the loop between the second and the third cysteines in the molecule, and the functional P1 residue (arginine inside all peptides) is located within this loop. This P1 residue is well defined in its binding site within the S1 specificity pocket of trypsin. The result of the stable hydrogen-bonding interactions that surround the scissile peptide bond is slow hydrolysis immediately after the critical arginine residue (Bateman and James, 2011).

The first discovered α-hairpinin with trypsin inhibitory activity was isolated from buckwheat (*Fagopyrum esculentum*) seeds by Park et al. (1997). Two peptides, called BWI-2a and BWI-2b, were identified BWI-2b contains 51 aa, while BWI-2a lacks three amino acid residues at the C-terminal part of the molecule. The reactive sites of both peptides are located immediately after Arg-19, between the two CXXXC motifs. Both peptides have a specific α-hairpinins Cys-motif and showed no relatedness to the other trypsin inhibitors reported earlier (Belozersky et al., 1995). Surprisingly, the BLASTP search revealed significant homology of these peptides with the N-terminal region of plant vicilin, the group of storage proteins widely distributed among dicots and monocots. The highest sequence similarity was observed between BWI-2b and the first exon of the cotton (*Gossypium hirsutum*) vicilin gene (Chlan et al., 1986), sharing 14 identical residues and possessing the same Cys-motif. The author speculated that BWI-2b might be processed from the first exon of the ancestral vicilin gene (Park et al., 1997).

BWI-2c was the third trypsin inhibitor belonging to α-hairpinins isolated from buckwheat seeds. BWI-2c has a significant similarity to BWI-2b (∼63% identity) and completely blocks bovine trypsin at a molar ratio of 1:1 (Ki = 1.74 × 10^–10^M). The hydrolysis of the peptide during incubation with trypsin occurred between residues Arg-19 and Trp-20. Besides this activity, BWI-2c acted as a potent suppressor of the trypsin-like protease from caterpillars (*Galleria mellonella*). The 50% inhibition of the *G. mellonella* enzyme required an amount of BWI-2c 10-fold less than that for bovine trypsin. This peptide was not able to inhibit the activity of cysteine proteases from the larvae of beetles *Tribolium castaneum*, *T. molitor*, and *Blatella germanica* as well as the serine proteases chymotrypsin, elastase, and subtilisin (Oparin et al., 2012).

Another trypsin inhibitor that also belongs to the α-hairpinin family is C2 peptide, isolated from maturing pumpkin (*Cucurbita maxima*) seeds. C2 peptide contained 51 aa residues and differed from BWI-2b significantly since they share only 18% identical residues. Trypsin 10 (μg) was completely inhibited by 1.2 nmol of the C2 peptide. Presumably, the reactive site of pumpkin trypsin inhibitor was located between Arg-21 and Gly-22 (Yamada et al., 1999).

Ultimately, a VhTI trypsin inhibitor was discovered while screening seeds of Veronica hederifolia (the ivy leaf speedwell) (Conners et al., 2007). This peptide consisted of 34 aa residues and was characterized as an extremely potent inhibitor effective at subnanomolar concentration, since the authors estimated the apparent KD as less than 1 nM (Conners et al., 2007).

### α-Hairpinins With Ribosome-Inactivating Activity

Kimura et al. (1997) isolated 6.5k-arginine-glutamate-rich polypeptide (6.5k-AGRP) from sponge gourd (*Luffa cylindrica*) seeds. Interestingly, half of 6.5k-AGRP was presented in seeds in full-length form while the other half was truncated at the C-terminus (lack two amino acids). The amino acid alignment of 6.5k-AGRP with seed storage proteins revealed the striking similarity of the peptide with the hydrophilic N-terminal sequence of cacao (*Theobroma cacao*) and vicilins from buckwheat (*F. esculentum*) and soybean (*Glycine max*) seeds (Kimura et al., 1997). Nothing was reported about the biological activity of this peptide. However, after a couple of years, Li et al. (2003) isolated the peptide named Luffin P1 that had an identical amino acid sequence to 6.5k-AGRP but lacked two amino acids on the N- and C-terminal parts of the peptide (Li et al., 2003). It was concluded that Luffin P1 was the truncated analog of 6.5k-AGRP.

Luffin P1 showed a strong inhibitory activity on protein synthesis in the cell-free rabbit reticulocyte (IC_50_ = 0.88 ηM), and its mechanism of action was identical to the ribosome-inactivating protein trichosanthin, which was an rRNA N-glycosidase. At that time, Luffin P1 was the smallest peptide reported to have a translational inhibitory activity. Experiments with gel filtration chromatography showed that this peptide formed (by electrostatic interactions) homotetramers in a hydrophobic buffer and that this fold might be appropriate for its N-glycosidase activity (Li et al., 2003). Further assays using static light scattering in combination with size exclusion chromatography showed that, in a hydrophilic buffer, Luffin P1 was depolymerized to a homodimer (Ng et al., 2011). Combining these data and the analysis of N-glycosidase activity the polymerization of Luffin P1 was suggested to enhance the activity of the peptide, and its polymer might be the active form of enzyme.

Moreover, as well as some other ribosome-inactivating proteins (geranium anti-HIV protein 31 kDa, maize RIP, and trichosanthin), Luffin P1 was found to have anti-HIV-1 activity and was able to bind with HIV Rev Response Element in HIV-1 infected C8166 T-cell lines (Ng et al., 2011). These data indicate that Luffin P1 shows some cytotoxicity and is more active against HIV replication (Ng et al., 2011).

## Three-Dimensional Structure of α-Hairpinins

The α-hairpinin family includes various peptides with different kinds of biological activity and low amino acid sequence identity. These heterogeneous molecules have, however, one common feature, the Cys-motif, forming a unique signature of the family and, more importantly, providing a characteristic 3D-configuration. The spatial structures of all α-hairpinin peptides are represented by two α-helices oriented antiparallel and joined by a loop. Two CXXXC motifs are located in different α-helices, and the cysteine residues connected via Cys1-Cys4 and Cys2-Cys3 are brought closer to form disulfide bonds. The N- and C-terminal tails are unstructured. This α1-turn-α2 arrangement can be easily distinguished from the β-strand decorated plant AMPs, including thionins, defensins, and knottins (Tam et al., 2015).

**FIGURE 2:**
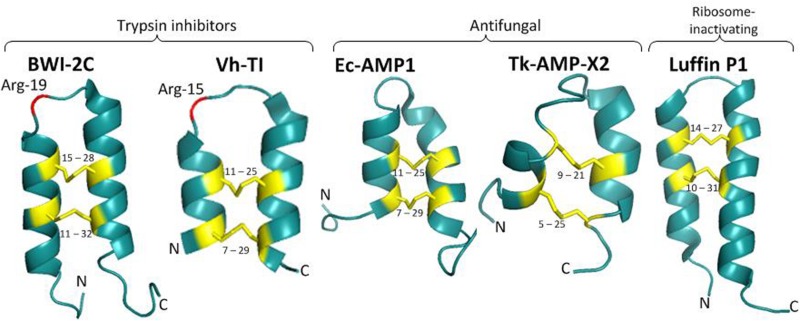
3D structures of α-hairpinins: BWI-2c (PDB code 2LQX), VhTI (PDB code 2PLX), EcAMP1 (PDB code 2L2R), Luffin P1 (PDB code 2L37), Tk-AMP-X2 (PDB code 2M6A). Disulfide bridges are shown as yellow sticks and the corresponding cysteine residue numbers are specified, crucial for trypsin inhibitors Arg residues are shown in red and specified.

The first spatial structure was obtained for the VhTI trypsin inhibitor in 2007 (Conners et al., 2007) using X-ray crystallography. It was determined that residues 7–15 and 18–29 were well ordered and formed two antiparallel α-helices connected with a loop and linked together by two disulfide bonds between residues Cys-7–Cys-29 and Cys-11–Cys-25 (Figure 2). The last six residues at the N-terminus and five at the C-terminus were disordered. In addition to native VhTI, a synthetic peptide lacking the disordered residues 1–4 and 32–34 was produced. Since VhTI was an extremely potent trypsin inhibitor, it was of great importance to resolve a crystal structure of complexes between trypsin and native or synthetic VhTI peptides. As a result, it was shown that each helix of both inhibitors forms a tightly associated bundle with the enzyme that was expected to provide overall rigidity. The α1-helix of VhTI was inserted into the trypsin recognition site at a steep angle; the crucial P1 (Arg-15) was situated in the S1 pocket of trypsin, P4 (Met-10) was placed in the enzyme S4 pocket, while P3 (Ala-13) was located in the S3 region. The α2-helix was situated away from the trypsin surface and connected by limited direct contacts with the enzyme; a Leu-21 side chain was inserted into the S1’pocket, and Glu-20 formed a hydrogen bond with the enzyme Tyr-39. In the complex between native peptide and trypsin, the scissile bond was hydrolyzed, and the cleavage occurred after the Arg15, while the active site of the enzyme (Ser-195) formedan acyl-enzyme covalent bond with the P1 carbonyl carbon. In contrast, the complex with the synthetic peptide remained intact, which clearly showed that VhTI could act as an inhibitor of trypsin in either its cleaved or uncleaved state (Conners et al., 2007).

Analysis of NMR spectroscopy of another trypsin inhibitor, the BWI-2c peptide, showed that its spatial structure was also represented by a pair of α-helices (Gln-5–Met-17 and Thr-22–Glu-34) connected with a loop and stabilized by two disulfide bonds (Cys-11–Cys-32 and Cys-15–Cys-28) (Figure 2; Oparin et al., 2012). The peptide also contained a short segment in a 3_10_-helix conformation (Lys-35–Glu-37) and a salt bridge with hydrogen made by the side chains of Arg-16 and Glu-29. The BWI-2c spatial structure was compared with the structure of VhTI. The crucial for inhibitory activity residues (Arg-15 in VhTI and Arg-19 in BWI-2c) had the same location in the spatial structure of both peptides. The canonical inhibitor loop between Met-17 and Thr-22 was functionally important for interaction with trypsin, and this structural element was very similar in both inhibitors (Otlewski et al., 2005). This loop was found to be quite flexible in the 3D structure of BWI-2c, which assumed the canonical conformation in peptide-trypsin complex, but in complex with VhTI, this loop was strictly fixed. The authors suggested that the mobility of the BWI-2c inhibitory loop in solution might indicate that the conformational space included the canonical conformation (Oparin et al., 2012).

The structure of the ribosome-inactivating Luffin P1 was determined by NMR spectroscopy (Ng et al., 2011). It also consisted of two anti-parallel α–helices (residues 5–17 and 22–39), which were bound by a short turn of four residues and stabilized by two disulfide bonds (Cys-10–Cys-31, and Cys-14–Cys-27; Figure 2). Four residues at the N-terminal tail and four residues at C-terminus were disordered. Luffin P1 had a positively charged unstructured N-terminus, the structured part with a positive and a negative charge, and the unstructured C-tail charged with one arginine and one glutamate residue. It should be noted that this structure diverged considerably from those of all previously described ribosome-inactivating proteins contained in the general large N-terminal domain with six α-helices and six β-sheets and small C-terminal domain with anti-parallel β-sheet and two α-helices with a bend in the middle. Li et al. (2003) observed that the C-terminus of Luffin P1 was similar to the arginine-rich motif (ARM) of viral Rev protein, which was shown to be responsible for exporting both unspliced and partially spliced HIV-1 mRNA from the nucleus to the cytoplasm. Moreover, both the α-hairpinin and ARM had a helix-turn-helix motif. It was suggested that Luffin P1 might inhibit HIV-1 replication by binding to the Rev Response Element and prevent Rev from transporting HIV-1 mRNA. In comparison with VhTI, Luffin P1 had more extended antiparallel α-helices. The authors suggest that Luffin P1 inhibits protein synthesis by selectively binding to some cellular components and that the recognition process is based on geometrical and charged complementarity (Ng et al., 2011).

The Tk-AMP-X2 spatial structure was resolved by NMR spectroscopy (Berkut et al., 2014). For this aim, the recombinant analog of this peptide and its ^15^N-labeled analog were produced in the *E. coli* expression system. The NMR data show that this short peptide had the typical spatial structure of α-hairpinins. Two α-helices (residues 4–8 and 15–24) were connected with a loop and short segment in a 3_10_ helix conformation (residues 9–11; Figure 2). The peptide was stabilized by two disulfide bonds between Cys-5 and Cys-25, Cys-9, and Cys-21. Interestingly, Tk-AMP-X2 had an entirely hydrophilic surface, suggesting that this α-hairpinin did not act in membrane disruption (Berkut et al., 2014).

Finally, the spatial structure of EcAMP1 was resolved. This consisted of two antiparallel (α-helices formed by the residues 7–14 and 22–30 that were stabilized by two disulfide bonds between Cys-7–Cys-29 and Cys-11–Cys-25 (Figure 2; Nolde et al., 2011). A type I (β-turn (residues 15–18) and a 310-helix turn (residues 19–21) linked the disulfide bonds together. Residues 1–6 at the N-terminal tail and 31–37 at C-terminal were unstructured; this suggests the possible formation of a transient hydrogen bond between the backbone amide of Cys-7 and a side-chain carboxylate group of Asp-37. EcAMP1 and VhTI had exactly the same location of cysteine residues (at positions 7, 11, 25, and 29) and proline residue (at position 19). However, the crucial Arg-15 residue responsible for recognition of VhTI by trypsin was absent in EcAMP1 (Figure 1). As a result, EcAMP1 displayed no inhibitory activity against trypsin and was cleaved as a normal substrate.

It is worth noting that the specific α-hairpinin cysteine pattern itself could not be used for classification and 3D-structure prediction. For example, Nolde et al. (2011) observed anintriguing similarity of this α-hairpinin fold and spatial structure of some scorpions and cone snail toxins (Nolde et al., 2011). The later molecules were also short (22–27 residues), and contained the specific α-hairpinin Cys-motif (X_n_C^1^X_3_C^2^X_n_C^3^X_3_C^4^X_n_) and the same the disulfide connectivities (Cys1–Cys4 and Cys2–Cys3). The peptides belonged to the group of potassium channel blockers and were isolated from different animals, in particular, κ-hefutoxins and OmTx1–3 was found in scorpions *Heterometrus fulvipes* and *Opisthacanthus madagascariensis*, respectively, and both flf14a–c and vil14a toxins were revealed in the cone snail *Conus floridanus floridensis* and *C. villepinii*, respectively. Moreover, the Cys-motif of EcAMP1, as well as Cys-motif of all α-hairpinins, was the part of specific Cys-motif of plant thionins. The evolutionary process of both plant families was unknown and arguable; however, if the short α-hairpinin motif was excised from the thionin fold, then this reduced α-hairpinin signature was responsible for biological activity and interaction with the main targets of action (Nolde et al., 2011).

## The Modular Structure of Genes Encoding α-Hairpinins

The gene structures of five α-hairpinins are known at present, and these can be divided into two types. The first type of gene encodes a precursor protein consisting of a signal peptide, a Cys-rich domain with 2–4 putative (α-hairpinins, and a hydrophobic domain with high similarity to vicilin seed storage protein. The second type consists of a signal peptide, a long multimodular cassette with 5–12 putative (α-hairpinins, and a short C-terminal domain with no homology to vicilins. During proteolytic processing, the long precursor protein is cleaved by various plant proteases into several mature (α-hairpinins.

**FIGURE 3:**
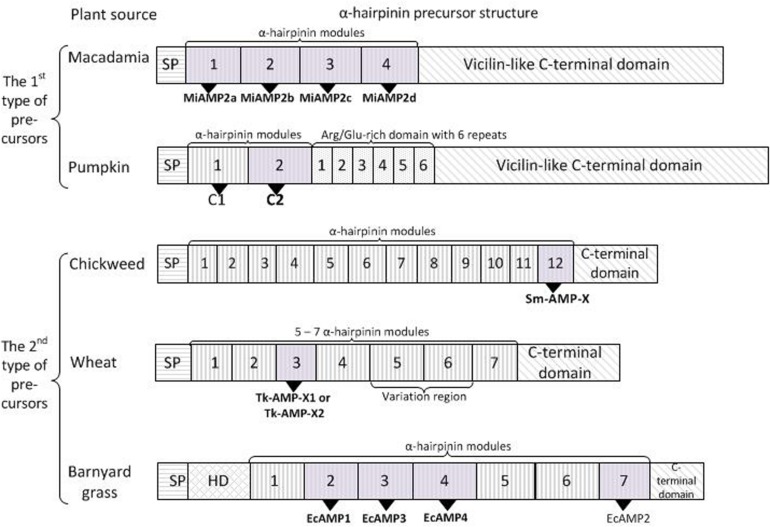
Two types of α-hairpinin precursors. The first type of precursors had C-terminal domain with similarity to vicilin seed storage peptide, while those of the second type shown no similarity. Signal peptides (SP) and C-terminal domains are boxed; HD – hydrophobic domain. Mature α-hairpinins with biological activity are shown as gray boxes and indicated in bold.

The first cDNA encoding α-hairpinin was obtained by Marcus et al. (1999), who isolated the MiAMP2c peptide from Macadamia integrifolia (Marcus et al., 1999). Three cDNA clones were detected while searching for the MiAMP2c precursor. All of them encoded a long precursor protein (more than 600 aa) consisting of an N-terminal signal peptide, a hydrophilic N-proximal region, and a C-terminal hydrophobic region (Figure 3). The hydrophilic region contains four segments with peculiar for (α-hairpinins Cys-motifs, and the exact MiAMP2c sequence was found in the third segment of clone 3. The revealed putative (α-hairpinins contained 35–50 aa residues and shared about 26% identity. The second and fourth segments were isolated from seed extracts and named MiAMP2b and MiAMP2d. Biological assays of the peptides have shown that they are active against plant fungal pathogens V. dahlia and Leptosphaeria maculans (Marcus et al., 1999). The attempts to identify the first putative peptide (MiAMP2a) failed; therefore, it was decided to express it in *E. coli* culture (Marcus et al., 1999). Using heterological expression, three recombinant peptides fused with a His-tag were obtained. The first peptide named His-MiAMP2c had activity similar to MiAMP2c. The second peptide was His-MiAMP2a, and this peptide was also active, mainly against *Botrytis cinerea* (IC_50_ = 8 μg/ml). The third peptide included a full cassette expressing all MiAMPs and displayed very little antimicrobial activity, with IC_50_ values of 32 and 64 μg/ml against *F. oxysporum* and *B. cinerea*, respectively. This finding suggests that processing of the precursor protein and obtaining the short peptide with the correct structure is vital for the biological activity (Marcus et al., 2008).

Interestingly, that C-terminal hydrophobic region of all MiAMP precursors had significant homology to several vicilins, 7S globulin proteins. In particular, the identity with pumpkin (*C. maxima*) vicilin was around 50%, and with cocoa (*T. cacao*) and cotton (*G. hirsutum*) vicilin proteins the amino acid identities were 43 and 45% respectively. Sequencing of a genomic clone encoding MiAMP2c cDNA revealed the presence of one intron-containing 203 bases located in the C-terminal hydrophobic region. This intron location exactly matched the first intron position in cotton and cocoa vicilin genes; the latter had four and five introns, respectively. Altogether, MiAMP2c was the first identified α-hairpinin released from the precursor, which contained the Cys-rich domain with four α-hairpinins and a hydrophobic domain with significant similarity to the vicilin seed storage protein (Marcus et al., 1999).

The described structure of cDNA is typical for several α-hairpinins. For example, the long precursor protein (PV100) accumulating in vesicles of maturing pumpkin seeds (*C. maxima*) was composed of a signal peptide followed by a hydrophilic cysteine-rich domain with two peculiar Cys-motifs, an Arg/Glu-rich domain composed of six homologous repeats, and a vicilin-like domain (Figure 3; Yamada et al., 1999). Two mature α-hairpinins, named C1 and C2, are located in the hydrophilic cysteine-rich domain and are cleaved from the precursor protein by specific vacuolar processing enzymes. The C2 peptide was shown to have a trypsin inhibitory activity. The authors also suggested that the PV100 precursor protein might be converted into different functional proteins, such as a proteinase inhibitor, Arg/Glu-rich peptides, and a vicilin storage protein (Yamada et al., 1999).

A remarkable structure of α-hairpinin precursors was observed for the Sm-AMP-X peptide, which was isolated from the seeds of the common chickweed (*Stellaria media*) (Slavokhotova et al., 2014b). Unlike the above mentioned α-hairpinin precursors, Sm-AMP-X precursors had no sequence similarity with vicilin storage protein precursors. Chickweed precursor protein contained an N-terminal signal peptide, a short C-terminal prodomain (merely 19 aa), and an elongated central part of the precursor, that was extremely cysteine-rich and contained as many as 12 tandem Cys-motifs typical for α-hairpinins (Figure 3). The last segment in this long cassette corresponded to the Sm-AMP-X peptide. The presence of another 11 peptides was confirmed by mass spectrometry of several chromatographic fractions obtained by stepwise purification of the acid seed extract. Besides, the single genomic DNA fragment that completely matched the sm-amp-x cDNA sequence, two pseudogenes were found. These contained stop codons in translated regions leading to significant truncation, and the cDNAs corresponding to these genes were not revealed. The authors speculate that during evolution, the original gene was subjected to several subsequent duplication events, resulting in one functional gene and one truncated pseudogene with a mutation inside the translated region. Interestingly, *sm-amp-x* cDNA is expressed in flowers and seedlings but not in stems, roots, or leaves (Slavokhotova et al., 2014b).

Another interesting structure of cDNA was described for Tk-AMP-X1 and Tk-AMP-X2 from the seeds of *Triticum kiharae* (Utkina et al., 2013). Seven genes encoding Tk-AMP-X peptides were isolated, six of which were expressed in immature seeds. All these genes contained no introns in their sequence and code for the precursors with the following structure: a short N-terminal signal peptide followed by a long cysteine-rich domain with characteristic α-hairpinin modules separated by linkers and finally a C-terminal prodomain (Figure 3). The observed precursors differed by the number of motifs in the cysteine-rich domain, in particular, four precursors that were designated as short, contained five α-hairpinin modules; one precursor had medium-size and consisted of six modules; and finally, two precursors were termed as long since they had seven α-hairpinin segments. In general, 27 novel α-hairpinin-like peptides were predicted, most of which were identified in seed extracts by mass spectrometry. Interestingly, the most variable were short peptides that differ by single substitutions and insertions/deletions, while sequences of long precursors were quite conservative. The distribution of *tk-AMP-X* gene homologs among *Triticum* and *Aegilops* species was also observed. As a result, it was discovered that five-modular precursors were associated with genome D originating from *Ae. tauschii*, six-modular precursors with genome A (*T. monococcum*, *T. boeoticum*, and *T. urartu*), and seven-modular precursors with genome B or the closely-related genome G. The analysis of cDNA expression revealed that fungal infection upregulated Tk-AMP-X genes, and abiotic stimuli (including elevated temperatures and high salt concentrations) also activated gene expression, while low temperatures downregulated gene expression (Utkina et al., 2013).

The precursor of EcAMP α-hairpinins isolated from barnyard grass had the same structure as the precursors of α-hairpinins from chickweed and wheat had. While searching for EcAMP cDNAs, more than 30 clones were obtained. However, because of the high GC content, that reached around 80% in the 5’-region, no full-length cDNAs or DNAs coding for the precise EcAMPs were found. The combined DNA fragments encoded one extended EcAMP precursor that consisted of a short N-terminal signal protein, a small hydrophobic domain followed by a long cysteine-rich cassette with seven α-hairpinin modules separated by 16–27 aa linkers, and ended with C-terminal prodomain (Figure 3; Ryazantsev et al., 2014). The second α-hairpinin module corresponded to EcAMP1, the third to EcAMP3, the fourth to EcAMP4, and the seventh to EcAMP2. Similar to chickweed and wheat α-hairpinin genes, the genes encoding EcAMPs were intronless. Interestingly, EcAMP cDNA was expressed only in seed embryos during a short period of maturation or in calluses, and this cDNA was not found in barnyard grass seeds, roots, leaves, or stems (Ryazantsev et al., 2014).

In conclusion, two types of α-hairpinin precursors have been described, and there is now great interest in the discovery of a third type of precursor, very popular for plant AMPs when the prepropeptide consists of a signal peptide followed by the only mature α-hairpinin and ended with a C-terminal domain. Due to the increasing amounts of plant transcriptome and genome data, it is now possible to predict novel AMPs and α-hairpinins using bioinformatic pipelines, rather than to isolate peptides by biochemical methods. It is easy to predict the first type of α-hairpinins that displays similarity to the vicilin protein. Even simple analysis of plant vicilins using BLAST showed that some of them (vicilins from cotton, cocoa, pumpkin, maize, barley, peanut, soybean) contain a Cys-rich domain with 1–3 α-hairpinin modules (Marcus et al., 1999). This approach was used for investigation of novel putative α-hairpinin from cacao that displayed similarity to MiAMPs from *M. integrifolia* and showed antifungal activity (see below).

The second type of α-hairpinin precursors has no significant similarity to vicilin storage proteins. Therefore, it is rather difficult to predict these using BLAST algorithms, and in this case, the peculiar Cys-motif that is obligate for all α-hairpinin peptidescan be used to search for novel putative AMPs. Several programs have been developed for *in silico* prediction of cysteine-rich and other defense peptides and proteins based on transcriptomic or amino acid sequences. For example, CS-AMPPred (Porto et al., 2012) predicts cysteine-stabilized AMPs, but it has very low specificity (Shelenkov et al., 2018); SPADA predicts cysteine-rich peptides without focus on particular function; and the recently developed Cysmotif searcher (Shelenkov et al., 2018) pipeline uses a revised set of precise cysteine motifs to search for various classes of cysteine-rich peptides, including defensins, lipid-transfer protein, hevein-like peptides, and others. Although these software tools are not specific to α-hairpinins, they can be used for preliminary revealing them in next-generation sequencing data. For example, 17 and 18 putative α-hairpinins were found in *Leymus arenarius* and *S. media* transcriptomes, respectively, using the Cysmotif searcher pipeline (Slavokhotova et al., 2015, 2017b). Also, since α-hairpinins possess a signature amino acid sequence, it is possible to identify them using software dedicated to the detection of a specific signal or regularity in nucleotide or amino acid sequences (Shelenkov et al., 2018). However, *in silico* searching for novel α-hairpinins still represents a great challenge for algorithm developers. The first successful example of such a prediction is described in the next section.

## α-Hairpinins in Biotechnology

α-Hairpinins represent short molecules with a rather simple tertiary structure that makes the peptides promising for biotechnology approaches. Currently, a genetic engineering approach is used to investigate the crucial residues of known peptides, to express various peptides based on bioinformatic prediction, and to obtain the peptides possessing novel properties beneficial in medicine and agriculture.

Marcus et al. (2008) observed that vicilins from cocoa (*T. cacao*), cotton (*G. hirsutum*), and pumpkin (*C. maxima*) had a hydrophilic domain with some α-hairpinin-like segments and hydrophobic domain with the seed storage function. The authors wonder whether other vicilin-derived peptides with peculiar Cys-motif also exhibited antimicrobial activity *in vitro*. For this aim, they chose the precursor most similar to MiAMP2c, the precursor protein from cocoa vicilin, which has two α-hairpinin-like segments, termed TcAMP1a and TcAMP1b. Both of the predicted peptides were expressed in an *E. coli* system, and the recombinant peptides with His-tag were tested against the range of plant pathogens. As a result, His-TcAMP1b appeared to show strong antimicrobial activity with IC_50_ values of 16 μg/ml against plant pathogenic fungal species *Aschochyta rabiae* and *F. oxysporum*, while His-TcAMP1a did not show any activity and inhibited *A. rabiae* at concentrations up to 64 μg/ml. The obtained results presented a novel efficient method for testing peptide fragments predicted from vicilin protein sequences for their potential antimicrobial activity (Marcus et al., 2008).

A similar genetic engineering approach was used for the functional study of crucial amino acid residues of MBP-1 peptide from maize (*Z. mays*) kernels. Sousa et al. (2016) created two variants of MBP-1 to identify the crucial elements of the molecule related to the antibacterial activity (Sousa et al., 2016). In the first mutant, Var 1, the tryptophan residue at the center of the hydrophobic core was replaced to alanine (Trp-20?Ala-20) to study the influence of hydrophobicity to bactericidal activity. In the second variant (Var 2), all cysteine residues were replaced with alanines to investigate the role of disulfide bridges in biological activity. Since 50 μM of MBP-1 induced near to total inhibition of *E. coli* DH5-α, the activity of α-hairpinin mutants were also tested against this bacterium, but no effect was observed for either of the peptides until a concentration of 400 μM. Considering that several plant AMPs act toward microorganisms by membrane disturbance, the effect of MBP-1 and Var 1 on *E. coli* membranes was investigated. As a result, none of the peptides seem to have membrane disturbance at MIC levels and double MIC levels (100 μM). However it was shown that some AMPs could arrest the growth of bacteria by binding its DNA and inhibiting the transcription and translation process without membrane permeabilization (Haney et al., 2013). To check whether MBP-1 and the mutants had DNA-binding activity, these peptides were evaluated in a gel retardation assay. It was found that MBP-1 could indeed bind the plasmid DNA if the concentration exceeded 3.12 μM, while Var1 and Var2 acted only at concentrations higher than 25 μM. The results of this experiment suggested that MBP-1 had a DNA-binding activity and might inhibit the transcription or translation processes in bacteria (Sousa et al., 2016).

The spatial structures for MBP-1 and its two mutants were modeled *in silico*. While the 3D structure of Var2 was generated *ab initio*, the structure of MBP-1 and Var1 were constructed based on the EcAMP-1 structure, because MBP-1 displayed up to 60% of identity to EcAMP1. For all three structures, a helix-turn-helix motif was peculiar, including the Var2 mutant without cysteine residues and disulfide bonds. The surfaces of all peptides were positively charged, in particular, Var 2, which possessed the most cationic surface, followed by MBP-1 and Var 1. The solvent-accessible surface area and the radius of gyration were higher in Var2 structure than that of Var1 and MBP-1, showing that the latter structures were more compact. Comparison of MBP-1 and Var1 using an essential dynamics method revealed that the MBP-1 structure was more flexible and that it could also contribute to tighter interaction with DNA and finally led to the higher activity (Sousa et al., 2016).

Recently the amino acid sequence of a trypsin inhibitor (FtAMP) from Tartary buckwheat (*F. tataricum*) was derived from its DNA sequence (Cui et al., 2018; Table 1). This α-hairpinin showed high identity to BWI-2c and differed only by two additional amino acids at the N-terminus. *In silico* modeling of the FtAMP 3D structure revealed that the peptide consisted of two α-helices (Gln-8–Met-18; Thr-24–Glu-36) connected with two disulfide bridges between cysteine residues (Cys-13–Cys-34; Cys-17–Cys-30). Five amino acids (Met19–Ser23) at the central loop had a random coil conformation and represented the active region, situated on the surface of the inhibitor. Trypsin hydrolyzed FtAMP immediately after critical Arg-21. Two mutant recombinant peptides were produced by site-directed mutagenesis and were expressed in *E. coli* culture: the first peptide contained an alanine residue instead of the crucial Arg-21 (FtAMP-R21A) and the second had phenylalanine residue (FtAMP-R21F) in this position. The trypsin inhibitory activities of both mutants were significantly reduced and were around 15% in comparison with wild-type FtAMP. However, they acquired inhibitory activity against elastase and α-chymotrypsin, while the native FtAMP showed no inhibitory effect. Interestingly, all three peptides displayed antifungal activity against *Trichoderma koningii*, *Rhizopus* sp., and *F. oxysporum*, with MIC values of 8 and 16 μM, respectively, suggesting that the mutations in the inhibitory site did not affect antifungal activity (Cui et al., 2018).

### Using α-Hairpinin Scaffold for Drug Development

Genetic engineering methods were used to obtain a mutant α-hairpinin molecule possessing novel properties beneficial in medicine. Berkut et al. (2014) observed that the Tk-AMP-X2 peptide shared structural similarity to potassium channel blockers κ-hefutoxin 1 from *Heterometrus fulvipes* (Srinivasan et al., 2002) and OmTx1–3 from *Opisthacanthus madagascariensis* (Chagot et al., 2005). Although the listed molecules had a low identity, the dyad crucial for the κ-hefutoxin-1 function was revealed (Tyr-5 and Lys-19). According to the “functional dyad” concept, it is believed that two highly conserved residues (one lysine and the other may be tyrosine, phenylalanine, or leucine) are involved in ligand-receptor interaction and provides potassium channel blockage (Gasparini et al., 1998; Shakkottai et al., 2001). The corresponding substitutions were made at the TkAMP-X2 sequence, in particular, the replacement Glu/Tyr-6 and Met/Lys-22. An additional substitution (Lys/Glu-23) was performed in order to avoid the positive charge at this position that might significantly diminish the activity. At 40 μM, the native Tk-AMP-X2 peptide and its mutant (Tk-hefu) were assayed as part of a screening panel of Kv channels, including members of the Shaker, Shab, Shaw, and erg families. Tk-AMP-X2 was not active against the tested channels, while Tk-hefu selectively targets members of the Shaker family (Kv1.2, Kv1.3, and Kv1.6). The constructed concentration-response curve displayed that the IC_50_ of Tk-hefu to Kv1.3 channels was 34 ± 2.8 μM. It was also found that Tk-hefu inhibited Kv1.3 channels in a dose-dependent, but also voltage-independent manner, and the activity of Tk-hefu was slightly greater than that of the dyad donor κ-hefutoxin 1. Note that there are some toxins, in particular members of the κ-KTX family that, similar to Tk-hefu, do not modify the activation kinetics of Kv1.3 channels. The authors suggested that non-conservative residues varied between κ-hefutoxin 1 and other members of the family might be responsible for the modulation of Kv1.3 channels (Berkut et al., 2014).

In subsequent work, Berkut et al. (2019) resolved the spatial structure of Tk-hefu using NMR spectroscopy and redesigned the surface of this molecule to better match the surface of the channel pore (Berkut et al., 2019). It was shown that Tk-hefu differed from the parent Tk-AMP-X2 by having more tightly coiled α-helices that were almost antiparallel to each other (∼160°), while the native molecule had crossed α-helices at angles of ∼130° and was less stable. This modification occurred because of the presence of Tyr-6, which form a π-cation contacted with lysine residues from the C-terminal helix. The obtained NMR structure of Tk-hefu was used for modeling its complex with the target channel, and Kv1.2/2.1 paddle chimera channel complex with charybdotoxin (ChTx) (Banerjee et al., 2013) was chosen as a template. The crucial amino acid residues for interaction with the channel were Tyr-6 and Lys-22, which is in good agreement with the “functional dyad” concept. The spatial superimposition of the Tk-hefu dyad with the classical dyad of ChTx resulted in the following model, where Lys-22 stuck to the selectivity filter, and Tyr-6 fixed the ligand against the channel vestibule. Using computational observation of Tk-hefu-Kv1.3 surface interactions, the negative impact of Glu-23 was revealed and indeed complex with Tk-hefu-2 molecule where Glu-23 was reversed to initial Lys showed a favorable change in the interaction energy profile. Electrophysiological assays confirmed that, in comparison to Tk-hefu that inhibited Kv1.3 channels with the IC_50_ value of 31.3 ± 5.1 μM, Tk-hefu-2 had an IC_50_ of 2.3 ± 0.4 μM, meaning that Tk-hefu-2 not only retained Kv1.3 selectivity but also displayed ~20-times greater activity compared to Tk-hefu. Inhibition of Kv1.3 channels occurred rapidly, and Tk-hefu-2 binding was reversible (Berkut et al., 2019).

To test the importance of every dyad residue, two further mutants were expressed in *E. coli* culture and purified: Tkhefu-3 with substitution Lys/Met-22 and Tk-hefu-4 with Tyr/Glu-6. Computational analysis of interaction energy of the Tk-hefu-4-Kv1.3 complex showed unfavorable changes in the energy profiles, while the energy contribution of Met-22 in Tk-hefu-3-Kv1.3 was negligible. The obtained results were justified by electrophysiological assays that demonstrated the IC_50_ values of 12.9 ± 1.8 μM for Tk-hefu-4 and absolutely loss of function for Tk-hefu-3 that could not inhibit any of the tested channels at concentrations up to 100 μM. Concluding, Tk-hefu-2 was a potent inhibitor of the Kv1.3 channel with an activity 20-times greater than the initial Tk-hefu; both amino acid residues in the dyad were crucial for interaction with the channel since the reverse substitutions led to a decrease or complete loss of activity (Berkut et al., 2019). The results of this work clearly showed that even a simple scaffold, such as a helix-loop-helix fold in α-hairpinin, could be rationally engineered to perform novel molecules with perspective features for drug design.

## Conclusion

α-Hairpinins represents a small family of short cysteine-rich peptides with various modes of action. Most of these molecules have antifungal and antibacterial activity, while there are some members with trypsin inhibitory and ribosome inactivation activities. The amino acid sequences of α-hairpinins have a low identity to each other, and only cysteine residues are conservatively forming the peculiar Cys-motif. cDNAs encoding α-hairpinins might consist of a domain with several α-hairpinin segments and a vicilin domain, or might be presented as a long cassette including numerous α-hairpinin modules followed one by one. Unlike the diverged primary structure of α-hairpinins, the spatial structure is quite conservative and contains a pair of α-helices connected with a loop and stabilized with two disulfide bonds. This compact and relatively simple fold can be easily modified to obtain molecules with novel characters beneficial for biotechnology and medicine.

## Author Contributions

AS designed the review and wrote the first draft of the manuscript. ER reviewed and edited the manuscript. Both authors contributed to manuscript revision and read and approved the submitted version.

## Conflict of Interest

The authors declare that the research was conducted in the absence of any commercial or financial relationships that could be construed as a potential conflict of interest.
